# *In vitro* effect of uremic serum on barrier function and inflammation in human colonocytes

**DOI:** 10.1590/2175-8239-JBN-3949

**Published:** 2018-06-18

**Authors:** Laila Santos de Andrade, Maria Aparecida Dalboni, José Tarcisio Giffoni de Carvalho, Caren Cristina Grabulosa, Natalia Barros Ferreira Pereira, Danilo Takashi Aoike, Lilian Cuppari

**Affiliations:** 1 Universidade Federal de São Paulo, Programa de Pós-Graduação em Nutrição, São Paulo, SP, Brasil.; 2 Universidade Federal de São Paulo, Departamento de medicina, Divisão de Nefrologia, São Paulo, SP, Brasil.

**Keywords:** Uremia, Intestine, Large, Renal Insufficiency, Chronic, Uremia, Intestino Grosso, Insuficiência Renal Crônica

## Abstract

**Introduction:**

In chronic kidney disease (CKD), it has been suggested that alterations within the gut are associated with an inflammatory state and uremic toxicity. Studies suggest that uremia may impair the function of the intestinal barrier via the promotion of increased intestinal permeability. To understand the mechanisms that are involved in intestinal barrier damage in the setting of uremia, we evaluated the *in vitro* effect of uremic serum on transepithelial electrical resistance (TER), inflammation, and apoptosis in intestinal epithelial cells (T84).

**Methods:**

Pools of serum from healthy individuals, patients not on dialysis, and patients on hemodialysis (Pre-HD and Post-HD) were prepared. T84 cells were incubated for 24 h in medium, of which 10% consisted of the pooled serum from each group. After incubation, the TER was measured and the following parameters were determined by flow cytometry: expression of toll-like receptors (TLRs), production of reactive oxygen species (ROS), and apoptosis. The level of IL-6 in the culture supernatant was determined by ELISA.

**Results:**

No difference was observed among the groups with respect to TER, apoptosis, and ROS or the expression of TLR-2, TLR-4, and TLR-9. IL-6 secretion was higher (*p* < 0.001) in cells that were incubated with pre- and post-HD serum.

**Conclusion:**

The results that were obtained from this model suggest that uremic serum *per se* does not seem to impair the integrity of intestinal epithelial cells. The increased IL-6 secretion by cells that were incubated with HD serum suggests a potential effect of uremia in the intestinal inflammatory response.

## RESUMO

**Introdução:**

Tem sido sugerido que na doença renal crônica (DRC) a uremia pode causar alterações intestinais, tais como modificações na microbiota e danos à barreira intestinal, e que estas possíveis alterações podem ter uma relação importante com o estado inflamatório e a toxicidade urêmica apresentadas por pacientes com DRC.

**Objetivos:**

Avaliar o efeito *in vitro* do soro urêmico sobre a permeabilidade da monocamada de células epiteliais do intestino, inflamação e apoptose.

**Métodos:**

Pools de soro foram preparados a partir de soros de indivíduos saudáveis, pacientes em tratamento conservador e em hemodiálise (Pré e Pós-HD). As células T84 foram incubadas por 24 horas com os diferentes pools. Em seguida a TER foi medida e as células foram submetidas às seguintes análises: apoptose, produção de espécies reativas de oxigênio (EROs) e expressão de receptores toll-like (TLR) por citometria de fluxo e detecção de IL-6 no sobrenadante da cultura por ELISA.

**Resultados:**

Não foram encontradas diferenças, entre os grupos, com relação a TER, apoptose, EROs e expressão de TLR-2, TLR-4 e TLR-9. Já a secreção de IL-6 foi maior (*p* < 0,001) pelas células incubadas com soro pré-HD e pós-HD.

**Conclusão:**

Os resultados obtidos a partir deste modelo sugerem que a uremia *per se* parece não comprometer a integridade das células epiteliais do intestino. O aumento da secreção de IL-6 pelas células incubadas com soro HD (pré e pós) sugere um potencial efeito da uremia sobre a resposta inflamatória intestinal.

## INTRODUCTION

The gastrointestinal tract functions as a barrier between the external environment and the internal milieu of the body. The epithelial layer of the gastrointestinal tract forms a regulated, selectively permeable barrier that permits the passive entry of nutrients, ions, and water and simultaneously restricts the entry of pathogens into the underlying tissue compartments. Several physiological and pathological stimuli dynamically regulate the permeability of this epithelium by changes in structures that are involved in the mechanisms of cell adhesion and the formation of cellular junctions^1^^,^ 2.

In addition, intestinal epithelial cells (IECs) express numerous receptors and proinflammatory mediators that allow them to communicate with the immune system^3^^,^ 4. The IECs are the first line of defense against pathogenic luminal microbiota and play an important role in the tolerance of the gut lumen towards commensal microorganisms^4^^-^ 6. Via toll-like receptors (TLRs), the intestinal epithelial cells recognize bacterial cell components^7^^,^ 8. The TLRs trigger immunologic responses against pathogens but are also regulated to limit the inflammatory response to commensal microbiota in the lumen^5^^,^ 6. Moreover, the activity of TLRs seems to result in the reorganization of the structure of tight junctions, which favors epithelial barrier function^9^. Therefore, the recognition of commensal microbiota by TLRs is important in the maintenance of intestinal homeostasis^10^^,^ 11.

Chronic kidney disease (CKD) is a condition that is characterized by a gradual loss of kidney function overtime with consequent retention of a number of compounds collectively termed uremic toxins. In individuals with CKD, the presence of inflammation, which is an important mediator of disease-associated complications, especially in the cardiovascular system, is common^12^^,^ 13. Several factors contribute to the inflammatory state including the decreased clearance of proinflammatory cytokines, uremic toxicity, oxidative stress, metabolic acidosis, and the dialysis process itself^14^. Recently, it has been suggested that alterations observed in the composition of the gut microbiota^15^^-^ 17 and in intestinal permeability^18^^-^ 21 due to CKD may also contribute to the inflammatory response^18^^,^ 22 ^,^ 23.

Therefore, there has been a growing interest in the investigation of the role of uremia on intestinal permeability. Vaziri *et al*.^24^ conducted an *in vivo* study with uremic rats and observed a large reduction in the expression of tight junction proteins, such as claudin-1, occludin and zonula occludens (ZO)-1, in the colonic mucosa; this indicates marked damage to the intestinal barrier. Similar findings in the expression of those proteins were also found by the same group of researchers in an *in vitro* study of human colonocytes that were incubated in medium containing plasma from patients undergoing hemodialysis. Cells that were incubated in uremic plasma showed significant reductions in the expression of tight junction proteins, which was accompanied by a decrease in the transepithelial electrical resistance (TER); these events indicate an increased permeability of the monolayer cells and suggest that compounds present in uremic plasma may be involved in the process of intestinal damage caused by uremia^25^.

Because the mechanisms that are involved in intestinal barrier damage by uremia are not fully understood, we aimed in this *in vitro* study to investigate the effect of uremic serum on intestinal epithelial permeability and to evaluate whether uremic serum impacts the expression of TLRs, oxidative stress, and the secretion of proinflammatory cytokines in human colonic epithelial cells.

## METHODS

### SERUM POOL PREPARATION

Four pools of serum were prepared from the following groups: four healthy individuals, who served as controls (CTL), five non-dialysis-dependent patients with chronic kidney disease (NND-CKD; estimated glomerular filtration rate between 15 and 29 mL/min), and five patients on maintenance hemodialysis [before (Pre-HD) and after (Post-HD) a hemodialysis session]. Patients were not included if they were younger than 18 years or older than 70 years, underwent dialysis for less than 3 months, had diabetes *mellitus*, infectious or inflammatory diseases, HIV, cancer, or autoimmune diseases, used corticosteroids or immunosuppressants or had previously received a kidney transplant. All blood samples were collected under fasting conditions (8 hours) with the exception of the post-HD samples. After blood collection, the serum was immediately separated, and the pools of serum were prepared and stored at -80°C. The concentrations of creatinine, urea, parathyroid hormone, calcium, phosphorus, and potassium were determined in each pool of serum. The Human Investigation Review Committee of the Federal University of São Paulo approved the study, and informed consent was obtained from each subject.

### CELL CULTURE AND INCUBATION STUDIES

T84 cells were obtained from American Type Culture Collection (Manassas, VA, USA) and were grown in 75-cm^2^ tissue culture flasks in DMEM/F12 medium (Life Technologies Inc., Carlsbad, CA, USA). The medium was supplemented with sodium bicarbonate 1.2 g/L, L-glutamine 2.5 mM, HEPES 15 mM, sodium pyruvate 0.5 mM, 10% fetal calf serum, and 0.5% penicillin/streptomycin (10,000 IU/mL and 10 mg/mL, respectively), and the cells were maintained at 37°C in a humidified 5% CO_2_ incubator.

To establish a cell culture system of a polarized monolayer, the cells were grown in 12-well plates with Millicell Hanging Cell Culture Inserts with a 12-mm diameter and a 0.4-µm pore size (EMD Millipore Inc., Billerica, MA, USA) at 400,000 cells/insert. To qualitatively determine whether the T84 cells had reached confluence, formed tight junctions, and established cell polarity, the transepithelial electrical resistance (TER) across the monolayer was monitored using a Millicell ERS-2 Meter (EMD Millipore Inc., Billerica, MA, USA). The T84 monolayers were maintained for approximately 10 days in complete medium. The medium was changed every other day, and the TER was measured regularly. When the TER exceeded 1,000 Ω/cm², the monolayers were incubated for 24 h in DMEM/F12 medium of which 10% was the serum from each pool (CTL, NND-CKD, Pre-HD and Post-HD). At the conclusion of the 24-hour incubation period, the TER was measured. Then, the cells were processed for flow cytometric analysis, and the supernatants were centrifuged for 5 minutes at 4°C; the cell-free supernatants were stored at -80°C until they used for the cytokine analysis. Nine experiments were performed, and the results were used in the statistical analysis.

### EXPRESSION OF TLR-2, TLR-4, AND TLR-9

After incubation for each condition described previously, the cells were washed with PBS and trypsinized. To detect the expression of TLR-2, TLR-4, and TLR-9 on the cell surface, 1 × 10^5^ T84 cells were incubated in the dark for 15 min at room temperature with the corresponding fluorescence-labeled antibodies: APC-conjugated anti-human TLR-2 (TL2.1, eBioscience, San Diego, CA, USA), PE-Cy7-conjugated anti-human TLR-4 (HTA125, eBioscience, San Diego, CA, USA), and PE-conjugated anti-human TLR-9 (EB72-1665, BD - Pharmingen, San Diego, CA, USA). The cells were then washed with PBS, the supernatant was discarded and the final pellet was resuspended in PBS. The detection of all antibodies was performed by a flow cytometer (FacsCanto I, BD Biosciences, San Diego, CA, USA). Forward and side scatters were used to gate T84 cells and to exclude cellular debris. The expression of TLR2, TLR4, and TLR9 were presented as mean fluorescence intensity peak (MFI) and percentage (%). The Fluorescence Minus One Control method (FMO control) was used to identify and establish gate cells and to control overlapping fluorophores.

### DETECTION OF INTRACELLULAR REACTIVE OXYGEN SPECIES (ROS)

Intracellular ROS levels were detected by the conversion of 2,7-dichlorofluorescein diacetate (DCFH-DA) (Sigma, St. Louis, MO, USA) into fluorescent 2,7-dichlorofluorescein (DCF) in presence of radical of oxygen^26^. Before incubation of DCFH-DA, the cells were washed with PBS and trypsinized. To detect ROS, 1 × 10^5^ T84 cells were incubated in the dark for 30 min at 37ºC with DCFH-DA at a concentration of 0.3 mM. Then, the cells were washed with PBS, the supernatant was discarded and the final pellet was resuspended in 3 nM EDTA. The detection of ROS was performed by a flow cytometer (FacsCanto I, BD Biosciences, San Diego, CA, USA). Forward and side scatters were used to gate T84 cells and to exclude cellular debris. The expression of ROS were presented as mean fluorescence intensity peak (MFI) and percentage (%).

### DETECTION OF IL-6

The concentration of IL-6 in the conditioned cell-free supernatants from the T84 monolayer cells was determined by an ELISA test kit (HS human IL-6 kit from R&D Systems, Minneapolis, MN, USA) according to the manufacturer's instructions.

### ANALYSIS OF CELL APOPTOSIS BY FLOW CYTOMETRY

After incubation, the cells were washed with PBS and trypsinized. To detect apoptosis and necrosis, 1 × 10^5^ T84 cells were incubated in the dark for 20 min at room temperature with fluorescein isothiocyanate (FITC)-annexin V and propidium iodide (BD - Pharmingen, San Diego, CA, USA). Early apoptotic cells were annexin V-positive and PI-negative, whereas late apoptotic cells were positive for both annexin V and PI, and necrotic cells were PI-positive and annexin V-negative^27^. The readings were performed in a flow cytometer (FACSCANTO I, BD Biosciences, San Diego, CA, USA). Forward and side scatters were used to gate T84 cells and to exclude cellular debris. The data are presented as percentage (%).

### STATISTICAL ANALYSIS

The data are presented as the mean and standard deviation (SD) or as the median and interquartile range, as appropriate. The General Linear Model (GLM) was used for comparisons of the groups, followed by the LSD analysis using SPSS^®^ software version 18.0 for Windows (SPSS Inc., Chicago, IL, USA). Statistical significance for all analyses was established at p values < 0.05.

## RESULTS

**Figure 1:**
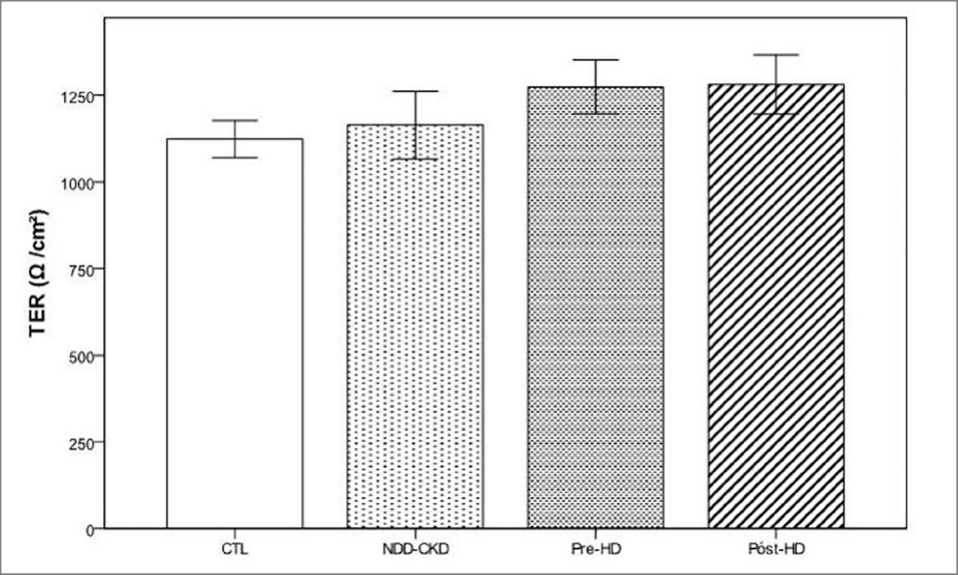
Transepithelial electrical resistance (TER) across the monolayer of T84 cells, after incubation under the different conditions. Data are presented as mean ± SE. *p* > 0.05 between groups. Serum pool: CTL- healthy control; NDD-CKD-non-dialysis-dependent chronic kidney disease; HD- hemodialysis.

Table 1 shows the laboratory data from each pool of serum that was used in the experiments. The values are in accordance with those that were expected for each condition. As seen in Figure 1, the uremic serum from the three different conditions did not promote changes in the transepithelial electrical resistance (TER) compared with the CTL or compared with each other.

**Table 1:** Demographic data from patients included in each pool and laboratory data from each pool of serum that was used in the study

Variables	CTL	NDD-CKD	Pre-HD	Post-HD
Age (years)	31.4 ± 8.98	57.4 ± 11.43	58 ± 9.67	-
Male [n (%)]	2 (40)	3 (60)	1 (20)	-
Creatinine (mg/dL)	0.82	2.9	11.54	3.33
Urea (mg/dL)	33	108	149	32.0
PTH (pg/mL)	23	161	763	381
Calcium (mg/dL)	9.7	9.2	9.0	10.5
Phosphorus (mg/dL)	3.4	4.1	4.8	2.3
Potassium (mEq/L)	3.8	4.7	4.8	2.8

Serum pool: CTL- healthy control; NDD-CKD- non-dialysis-dependent chronic kidney disease; HD- hemodialysis; PTH-parathyroid hormone.

**Figure 2:**
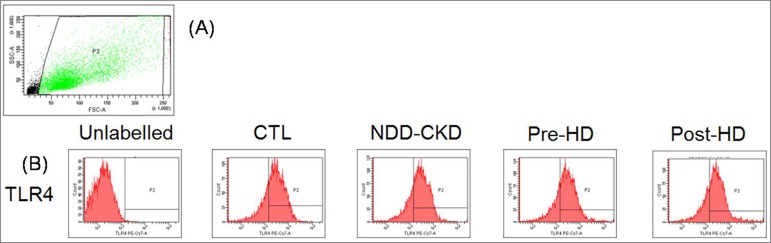
Illustration of the strategy performed to detect the expression of the toll-like receptors, production of ROS, and apoptosis by flow cytometry. (A) Forward and side scatters were used to gate T84 cells and to exclude cellular debris. (B) Example of Toll-like receptor 4 (TLR-4) expression in each condition of incubation. CTL- healthy control; NND-CKD- non-dialysis-dependent chronic kidney disease; HD- hemodialysis. TLR-4- Toll-like receptor 4.

**Figure 3:**
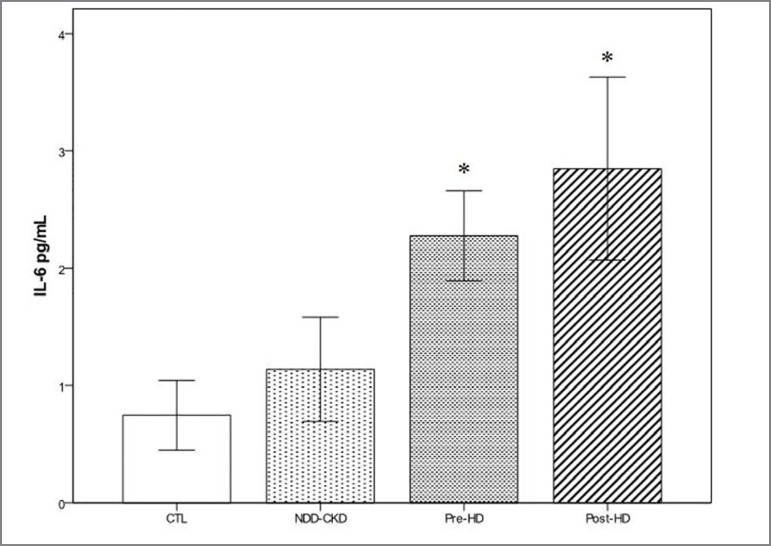
Production of IL-6 by T84 cells after incubation under the different conditions. Data are presented as mean (95% CI). * *p* < 0.01 *vs*. CTL and NDD-CKD. Serum pool: CTL- healthy control; NND-CKD-non-dialysis-dependent chronic kidney disease; HD- hemodialysis.

In addition, as shown in Table 2, the uremic serum from the three conditions did not promote changes in the expression of TLR-2, TLR-4 or TLR-9, or in the production of ROS compared with the CTL, as well as compared with each other. Figure 2 illustrates the strategy performed to detect the expression of these proteins and the production of ROS by flow cytometry. On the contrary, a higher secretion of IL-6 was found in cells incubated with pre- and post-HD serum compared with the CTL and NDD-CKD incubation conditions (Figure 3).

**Table 2:** Expression of TLR-2, TLR-4, and TLR-9 and production of ROS and IL-6 in T84 cells

Variables	CTL	NDD-CKD	Pre-HD	Post-HD	*p*
TLR-2 (MFI)	10,227 ± 2,653	10,391 ± 3,266	11,826 ± 3,422	14,940 ± 9,032	0.493
TLR-2 (%)	94.6 (89.9-96.9)	93.3 (85.1-97.7)	94 (88.4-98.2)	94.8 (87.2-96.8)	0.827
TLR-4 (MFI)	6,030 (5,537-8,727)	6,504 (5,609-8,364)	7,668 (6,263-9,834)	9,119 (5,743-15,812)	0.418
TLR-4 (%)	82.6 (76.2-91)	80.6 (76-92.5)	82 (77.2-93.4)	84.9 (78.8-91)	0.851
TLR-9 (MFI)	3,773 (2,149-9,303)	2,995 (2,115-12,856)	2,789 (2,111-7,273)	3,580 (2,980-8,425)	0.937
TLR-9 (%)	4.8 (1.15-7.7)	4.6 (1.07-8.72)	4.15 (0.82-6.8)	4.45 (1.15-8.65)	0.971
ROS (MFI)	293.4 ± 72.7	289.2 ± 54.83	291 ± 65.2	294.6 ± 44.8	0.999
ROS (%)	40.8 (34.5-65.7)	46.2 (35.9-62.8)	38.4 (32.3-57.5)	33.6 (25.3-50.5)	0.628
IL-6 (pg/mL)	0.76 ± 0.35	1.14 ± 0.53	2.27 ± 0.46*	2.85 ± 0.93*†	< 0.001

Data are presented as mean ± SD or as the median (interquartile range). *p < 0.01 vs. CTL and NDD-CKD; † *p* = 0.07 *vs*. Pre-HD. MFI- Mean fluorescence intensity; TLR-2- Toll-like receptor 2; TLR-4- Toll-like receptor 4; TLR-9- Toll-like receptor 9; IL-6- Interleukin-6. Serum pool: CTL- healthy control; NDD-CKD- non-dialysis-dependent chronic kidney disease; HD- hemodialysis.

As depicted in Table 3, good cell viability (> 80%) was observed in each incubation condition, and the uremic serum did not promote changes with respect to apoptosis.

**Table 3:** Viability, apoptosis and cell necrosis

Variables	CTL	NDD-CKD	Pre-HD	Post-HD	*p*
Viability (%)	80.5 ± 5.2	82.9 ± 4.35	82.7 ± 4.8	82.1 ± 3.7	0.841
Early apoptosis (%)	6.8 ± 2.35	5.8 ± 1.6	5.5 ± 1.95	6.2 ± 2.3	0.751
Late apoptosis (%)	6.5 (6.1-8.4)	6.3 (5.4-7.4)	6.5 (5.7-7.4)	6.4 (6.0-8.3)	0.755
Necrosis (%)	4.6 (2.7-9.1)	3.3 (2.1-8.7)	3.9 (1.8-9.9)	5.0 (2.3-7.0)	0.984

Data are presented as the mean ± SD or as the median (interquartile range). *p* > 0.05 between groups. Serum pool: CTL- healthy control; NDD-CKD- non-dialysis-dependent chronic kidney disease; HD- hemodialysis.

## DISCUSSION

In the present *in vitro* study with intestinal epithelial cells, we showed that the uremic serum from the three conditions tested did not promote changes in transepithelial electrical resistance (TER), oxidative stress or in the expression of toll-like receptors. However, an increase in the secretion of IL-6 was observed in cells that were incubated with pre- and post-HD serum. As far as we know, only one study evaluated the effect of uremic plasma on the permeability of monolayer intestinal epithelial cells. In contrast to our findings, Vaziri *et al*.^25^ found that uremic plasma (pre- and post-HD) promoted a reduction in TER with a concomitant decrease in the expression of tight junction proteins in T84 cells. Although we have carefully followed the steps described in the methods of this previous study, we were unable to reproduce their findings. The underlying reasons for the discrepancy in the results are unknown, but a few differences in the study protocol might be implicated. First, we used a pool of serum for each incubation condition while Vaziri *et al*. reported the cells were incubated separately with plasma from five patients. Second, the criteria we used for the selection of the patients to prepare the serum pools may have differed in some respects from the criteria used by Vaziri *et al*. However, as depicted in Table 1, the laboratory parameters from the pools prepared in our study were consistent with those that were expected for each experimental group.

The lack of change in markers of intestinal cell integrity in our study indicates that the uremic serum may not have impaired the paracellular permeability if a direct effect was expected. Although the incubation period may have some influence on the intestinal permeability, we decided to use 24 h based on the previous mentioned study 25. However, before starting the experiments we also tested two other incubation periods (6 h and 48 h) but the results were similar to that obtained with 24 h period. More importantly, it should be considered that the damage to the intestinal epithelial cells by uremia might occur indirectly due to its effects in the intestinal environment that involve the increase of urea influx into the gastrointestinal tract. This favors an increase in the fermentation of nitrogenous compounds, which would generate a large number of products that may negatively affect the intestinal epithelium^28^^-^ 31. In fact, in an *in vitro* study with T84 cells incubated with urea plus urease (a bacterial enzyme), a large reduction in TER and in the expression of tight junction proteins was observed, which suggests the deleterious effect of this enzymatic activity on intestinal epithelial cells^32^. The alterations in the biochemical milieu may result in changes in the composition and in the metabolic activity of the gut microbiota^30^. Indeed, a study with CKD has demonstrated important modifications in the composition of the intestinal microbiota. These modifications are characterized by the expansion of bacterial families that possess urease as well as indole- and p-cresol-forming enzymes, and a reduction of families that possess butyrate-forming enzymes^16^. The important role of the intestinal environment in the preservation of intestinal permeability has been demonstrated in a recent study. A significant attenuation in the alterations of tight junction proteins in colonic tissue was found when uremic rats were treated with fermentable dietary fiber, which is known to have a beneficial effect on enzyme activities and on microbiota composition^33^. Therefore, the effect of uremia in the intestine seems to be complex and involves the interaction of both the intestinal cells and the microbiota. Thus, the lack of a negative impact of uremic serum in the present study might be attributed to the absence of microbiota.

Disruption of the inflammatory balance in the gut represents a potential factor that contributes to the damage of the intestinal barrier^34^. Because it has been demonstrated in several cell types that uremic toxins have a stimulatory effect on inflammatory pathways^35^^-^ 39, we hypothesized that such an effect might also occur in intestinal epithelial cells. Therefore, we investigated for the first time whether uremic serum would modify the expression of toll-like receptors (TLRs) and the production of IL-6. Despite the lack of change in the TLRs under the uremic conditions in our experiments, an increase in the secretion of IL-6 was observed when the cells were incubated with serum from patients on hemodialysis (HD). Apparently, the activation of TLR-2, TLR-4, and TLR-9 did not seem to be involved in the stimulation of IL-6 release by the uremic serum. Although not tested in our study, the observed increase in IL-6 might be a consequence of the stimulatory effect of other cytokines, such as TNF-α and IL-1, which are often present at higher levels in the serum of patients on HD^40^^,^ 41. This assumption is somewhat plausible if we consider that the production of IL-6 did not increase when the cells were incubated with uremic serum from patients who were not dependent on dialysis. Indeed, the presence of some degree of renal function in these patients may allow for, at least to some extent, the removal of uremic toxins including cytokines. On the contrary, the removal of several types of molecules such as cytokines is relatively inefficient in the hemodialysis process^42^. The increased IL-6 secretion by cells that were incubated with HD serum did not seem to cause deleterious effects on the integrity of the cell monolayer because no change in the TER was observed. However, it is important to consider that the potential damage to the barrier function, which is caused by disturbances in the inflammatory response, is complex and involves the relationships among the different cell types, particularly those from the immune system. Therefore, the experimental model used in the present study does not allow us to conclude whether IL-6 secreted by the epithelial cells contributes to the overall inflammatory process. This finding deserves to be investigated further to advance the current knowledge on the relationship among CKD, inflammation, and intestinal barrier function.

In conclusion, we showed that the uremic milieu did not affect the integrity of the intestinal barrier, the expression of TLRs or the production of reactive oxygen species. The HD serum stimulated the secretion of IL-6 by intestinal epithelial cells. Because uremia may affect intestinal homeostasis through different pathways, further studies are necessary to better understand the relationship between CKD and the gut.
